# High momentum two-dimensional propagation of emitted photoluminescence coupled with surface lattice resonance

**DOI:** 10.1038/s41377-025-01873-3

**Published:** 2025-06-20

**Authors:** Yeonjeong Koo, Dong Kyo Oh, Jungho Mun, Artem N. Abramov, Mikhail Tyugaev, Yong Bin Kim, Inki Kim, Tae Ho Kim, Sera Yang, Yeseul Kim, Jonghwan Kim, Vasily Kravtsov, Junsuk Rho, Kyoung-Duck Park

**Affiliations:** 1 Department of Physics, Pohang University of Science and Technology (POSTECH), Pohang, 37673 Republic of Korea; 2 Department of Mechanical Engineering, Pohang University of Science and Technology (POSTECH), Pohang, 37673 Republic of Korea; 3 Department of Electrical and Computer Engineering, Purdue University, West Lafayette, IN 47907 USA; 4 School of Physics and Engineering, ITMO University, Saint Petersburg, 197101 Russia; 5 Department of Biophysics, Institute of Quantum Biophysics, Sungkyunkwan University, Seoul, 16419 Republic of Korea; 6 Department of Intelligent Precision Healthcare Convergence, Sungkyunkwan University, Seoul, 16419 Republic of Korea; 7 Department of Material Science and Engineering, Pohang University of Science and Technology (POSTECH), Pohang, 37673 Republic of Korea; 8 Center for Van der Waals Quantum Solids, Institute for Basic Science (IBS), Daejeon, 37673 Republic of Korea; 9 Institute for Convergence Research and Education in Advanced Technology, Yonsei University, Seoul, 03722 Republic of Korea; 10 Department of Chemical Engineering, Pohang University of Science and Technology (POSTECH), Pohang, 37673 Republic of Korea; 11 Department of Electrical Engineering, Pohang University of Science and Technology (POSTECH), Pohang, 37673 Republic of Korea; 12 POSCO-POSTECH-RIST Convergence Research Center for Flat Optics and Metaphotonics, Pohang, 37673 Republic of Korea; 13 National Institute of Nanomaterials Technology (NINT), Pohang, 37673 Republic of Korea; 14 Department of Semiconductor Engineering, Pohang University of Science and Technology (POSTECH), Pohang, 37673 Republic of Korea

**Keywords:** Metamaterials, Nanophotonics and plasmonics

## Abstract

Dramatic fluorescence enhancement in two-dimensional (2D) van der Waals materials (vdWMs) coupled to plasmonic nanostructures has the potential to enable ultrathin, flexible, and high-brightness illumination devices. However, addressing the limitation of locally scattered small plasmon-enhanced areas remains challenging. Here, we present a 2D plasmonic enhancement of photoluminescence (PL) spanning nearly 800 μm^2^, enabled by surface lattice resonance (SLR) in a 2D vdWM-Au slot lattice hybrid. The Au slot lattice is designed and fabricated using Babinet’s principle and Rayleigh’s anomaly to maximize radiative decay rate and induce non-local photo-excitation in a MoSe_2_ monolayer. For emitted PL coupled with SLR, enhanced by up to 32-fold, we investigate its in-plane directivity and long-range propagation using angle- and space-resolved spectroscopic PL measurements. Our experiment reveals that a nearly 800 μm^2^ 2D luminescent sheet can be achieved regardless of the size of the MoSe_2_ crystal, even with a sub-μm^2^ flake. This work provides a new type of ultrabright, large-area 2D luminescent material, suitable for a range of optical illumination, communication, and sensing devices.

## Abstract (web summary)

We achieve a 2D plasmon-enhanced photoluminescence and long-range surface propagation of it via surface lattice resonance (SLR) in a vdWM-Au slot lattice.

## Graphical abstract

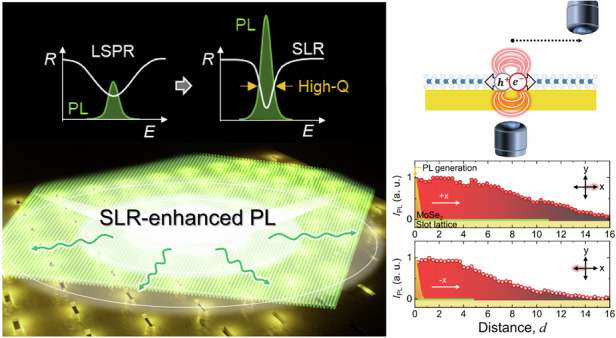

## Introduction

Since the first observation of direct bandgap photoluminescence (PL) in two-dimensional (2D) van der Waals materials (vdWMs), they have attracted significant attention as promising candidates for bright and energy-efficient illumination devices^1–3^. Recent advancements in the growth technology of vdWMs have enabled the fabrication of high-quality, large-area crystals, even reaching up to wafer scale^4–6^. In addition, with their atomically thin, sub-nanometer thickness, vdWMs exhibit an emission power per unit volume that far surpasses that of commercialized light-emitting devices. This extraordinary quantum property presents significant potential for next-generation ultrathin, foldable, large-area displays, especially for smart phones and watches^7–9^. Despite these advantages, the industrial application of vdWMs is hampered by their intrinsically low quantum yield, in contrast to successfully commercialized quantum dot light-emitting diodes (QLEDs) and organic light-emitting diodes (OLEDs)^10–13^.

Recently, to improve fluorescence quantum yield, vdWMs-plasmonic nanostructure hybrids have been extensively studied across various platforms^14–17^. By exploiting the Purcell effect in these hybrids, the radiative recombination rate of excitons is significantly increased, resulting in PL enhancement. However, since the Purcell-enhancement mechanism originates from the modified permittivity in nanostructured plasmonic metals and their localized plasmons, the local enhancement region is limited to just a few tens of nanometers squared^18^. Consequently, most vdWMs-plasmonic nanostructure hybrids cannot serve as effective surface sources of luminescence, failing to fully exploit the potential of 2D vdWMs. Moreover, in many cases, the field localization and maximum Purcell enhancement spots of plasmonic nanostructures are located on the embossed surfaces of metals, leading to unavoidable ohmic losses in vdWM emitters^19–21^. Therefore, a new type of vdWMs plasmonic nanostructure hybrid that enables large-area luminescence enhancement over a 2D surface with minimal energy loss is highly desirable.

In this work, we demonstrate a significant 2D plasmonic enhancement of PL emission over an area approaching 800 μm^2^, achieved through surface lattice resonance (SLR) in a vdWM-Au slot lattice hybrid. By employing Babinet’s principle and Rayleigh’s anomaly in the design of the Au slot lattice, we maximize the radiative decay rate, minimize ohmic energy loss, and induce non-local photo-excitation in a MoSe_2_ monolayer (ML). We observe up to a 32-fold enhancement in PL measurements and examine its in-plane directivity and long-range propagation through angle- and space-resolved spectroscopic PL measurements. Notably, our study demonstrates that a nearly 800 μm^2^ 2D luminescent sheet can be achieved regardless of the size of the MoSe_2_ crystal, even with sub-μm^2^ flakes. We believe that this work introduces a novel class of ultrabright, large-area 2D luminescent materials, with potential applications in optical illumination, communication, and sensing devices.

## Results

### MoSe_2_ ML-slot lattice hybrid for large-area PL enhancement

**Fig. 1:**
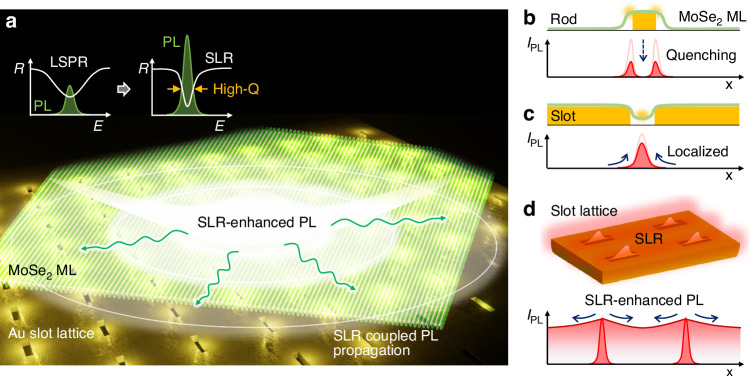
MoSe_2_ ML-slot lattice hybrid for large-area PL enhancement. **a** Conceptual illustration of SLR-enhanced PL of the MoSe_2_ ML on the Au slot lattice. **b**, **c** Comparison of the expected PL intensity (*I*_PL_) enhancement for a MoSe_2_ ML on a rod (**b**) and a slot (**c**) antenna. **d** Expected SLR-enhanced optical fields in a slot lattice for large-area PL enhancement

Figure 1a shows a conceptual illustration of a MoSe_2_ ML coupled to the Au slot lattice. The lattice structure with slot antennas of size 25 × 80 μm^2^ was prepared by e-beam lithography on an Au layer deposited onto a SiO_2_ substrate, and an exfoliated MoSe_2_ ML was subsequently transferred onto it. When we excite the antenna-vdWM hybrid with a linearly polarized continuous wave laser ($\lambda$ = 594 nm), strong field localization at each slot antenna is induced by the localized surface plasmon resonance (LSPR) effect. Subsequently, the scattering of localized fields at slot antennas produces diffracted waves on the 2D lattice plane. In addition, surface plasmon polaritons (SPPs) at the lattice produce strong surface fields through constructive interference. Hence, the resulting SPP-assisted SLR facilitates a drastic field enhancement over the 2D area^22,23^. This SLR then couples with the vdWM exciton PL and spreads out widely over the 2D surface, enabling a large-area PL enhancement with the local optical excitation. This is a distinctive advantage of the SLR-enhanced PL approach, compared to the previous local PL enhancement approaches using the LSPR effect of single plasmonic antennas.

In Fig. 1b–d, we show illustrations for the expected PL intensity (*I*_PL_) distributions on a MoSe_2_ ML coupled with a single rod antenna (b), a single slot antenna (c), and a slot lattice (d). Based on Babinet’s principle, the distribution of field localization shows complementary behavior for the rod and slot antenna^24–26^. For the rod antenna-coupled vdWM hybrid (Fig. 1b), the direct contact between the crystal and the metal surface at the antenna mode causes ohmic energy losses, giving rise to PL quenching^27,28^. By contrast, for the slot antenna-coupled vdWM hybrid (Fig. 1c), the localized optical field at the slot antenna mode can efficiently excite the excitons of the MoSe_2_ ML, leading to larger PL enhancement. Furthermore, by extending it into an optimal slot lattice (Fig. 1d), the emitted PL coupled with SLR is distributed over a wide 2D area.

### Design and characterization of a single-rod and slot antenna

**Fig. 2:**
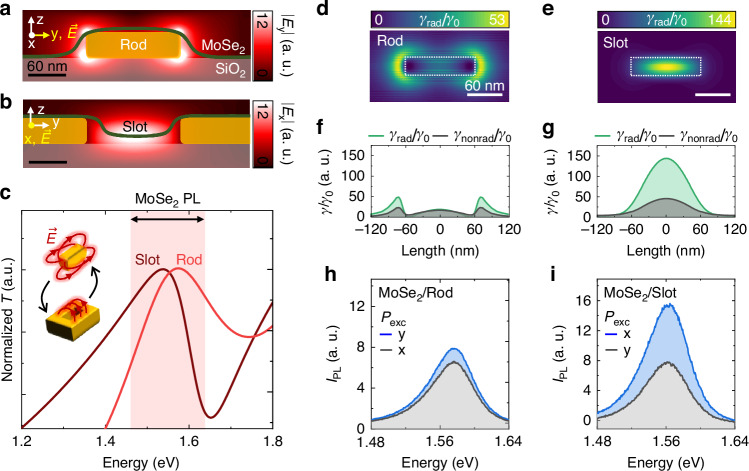
Design and characterization of a single-rod and slot antenna. **a**, **b** Simulated field distributions for the designed rod (**a**) and slot (**b**) antenna under the optimal excitation polarization (yellow). **c** Simulated transmittance spectra of the antennas exhibiting Babinet’s principle. **d**, **e** Simulated distribution of radiative decay rate enhancement ($\gamma$_rad_/$\gamma$_0_) for the in-plane emitters distributed at the rod (**d**) and slot (**e**) antenna. **f**, **g** Horizontal line traces of a radiative (green) and nonradiative (black) decay rate enhancement at the center of the rod and slot antenna, derived from (**d**) and (**e**), respectively. **h**, **i** Measured PL spectra of a MoSe_2_ ML coupled to the rod (**h**) and slot (**i**) antenna for different excitation polarization (*P*_exc_) conditions

Before the design and fabrication of the Au slot lattice, we first design, fabricate, and characterize the optimal single rod and slot antennas. Specifically, we design complementary rod and slot antennas with dimensions of 30 nm in width and 120 nm in length. The optimal height slightly varies, with the rod antenna set at 40 nm and the slot antenna at 30 nm. These dimensions are chosen to achieve spectral resonance, overlapping with the PL spectrum of a MoSe_2_ ML (1.48–1.64 eV). Figure 2a, b shows the simulated optical field distribution in the *yz* plane (*y*-axis along the longitudinal axis of both antennas) for the rod and slot antenna at the excitation photon energy of 1.96 eV. To maximize field enhancement, different excitation polarizations are set for each antenna. Specifically, polarization is aligned parallel to the longitudinal axis for the rod antenna and parallel to the transverse axis for the slot antenna. Following Babinet’s principle, the complementary rod and slot antennas exhibit a similar energy range in optical resonance (Fig. 2c), despite differences in their field distributions^24–26^. A slight deviation in resonance energy is observed due to the finite thickness of the rod and slot antennas, as well as the plasmonic effects of gold, causing deviations from the ideal conditions assumed by Babinet’s principle. However, the strong spectral overlap and complementary spatial distributions of the enhanced field suggest that Babinet’s principle remains robustly applicable to our system. The optical fields of the rod antenna primarily localize at the corners of the Au rod. Consequently, the close proximity of the metal surface to the 2D semiconductor can reduce the radiative decay rate due to ohmic energy losses. Conversely, the localized optical fields at the slot predominantly distribute across the suspended MoSe_2_ ML, facilitating effective coupling to excitons and resulting in a high radiative decay rate.

We then simulate the maps exhibiting the increased radiative decay rate ($\gamma$_rad_/$\gamma$_0_) for in-plane dipole emitters positioned near the rod and slot antennas, as shown in Fig. 2d, e (see Figure S1 for corresponding nonradiative decay rate ($\gamma$_nonrad_/$\gamma$_0_) maps). The emission energy of the emitters is set to 1.57 eV, tailored for exciton resonance within the MoSe_2_ ML. Line profiles of $\gamma$_rad_/$\gamma$_0_ (green) and $\gamma$_nonrad_/$\gamma$_0_ (black), derived from these maps, along the longitudinal axis of the antennas are shown in Fig. 2f, g. We confirmed that free-standing dipole emitters positioned at the slot antenna exhibit a quantum yield more than 2 times larger than those positioned at the rod antenna, attributed to smaller ohmic losses. For experimental validation, we acquire excitation polarization (*P*_exc_)-dependent PL spectra for the MoSe_2_ ML placed on the rod and slot antennas, respectively. Under optimal *P*_exc_ condition for each antenna (blue), the MoSe_2_ ML shows PL signals >2 times stronger when using the slot antenna compared to the rod antenna, as shown in Fig. 2h, i, owing to reduced PL quenching effects.

### Surface lattice resonance characteristics of the Au slot lattice

**Fig. 3:**
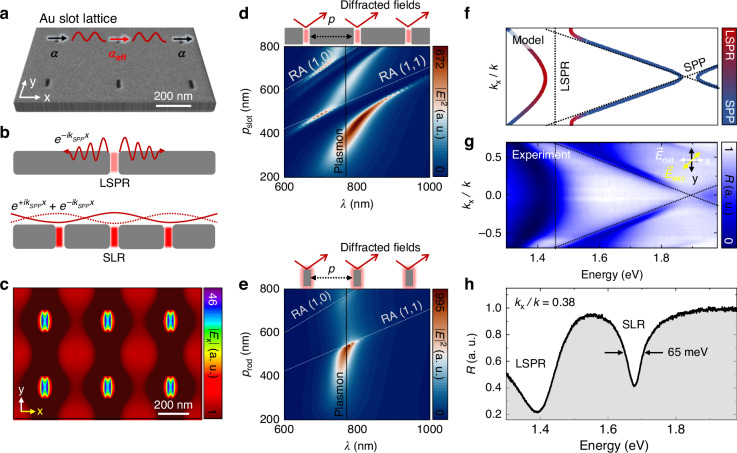
SLR characteristics of the Au slot lattice. **a** SEM image of the fabricated Au slot lattice. Effective polarizability $\alpha$_eff_ is induced by the dipole coupling of periodically arranged slot antennas, while each individual antenna has a polarizability $\alpha$. **b** Illustrations of LSPR and SPPs in a single slot antenna (top) and SLR in a slot lattice (bottom) resulted from the coupling between the LSPR and counterpropagating SPPs. **c** Simulated nonlocal field distribution on the slot lattice attributed to the SLR. *P*_exc_ aligns parallel to the *x*-axis (yellow). **d**, **e** Calculated field intensity as a function of the period (*p*) and wavelength ($\lambda$) for slot (d) and rod (e) lattices. Rayleigh’s anomalies (RAs) are indicated by white lines, and plasmon resonance is indicated by black lines. **f** Simulated SLR dispersion (solid lines) for the fabricated Au slot lattice, attributed to the coupling of SPPs (dotted lines) and LSPR (dotted lines). **g** Measured angle-resolved reflectance spectra for the slot lattice. Polarizations for excitation and detection on the slot lattice are indicated in the top-right corner. **h** Reflectance spectrum at *k*_*x*_/*k* = 0.38, extracted from Fig. 3g, showing sharp SLR and broad LSPR peaks

We then design, fabricate, and characterize the Au slot lattice to collectively couple the multiple slot antennas, eventually to exploit it for the large-area PL enhancement of the MoSe_2_ ML. Figure 3a shows the scanning electron microscope (SEM) image of the fabricated Au slot lattice with a period of 400 nm. When plasmonic antennas are arranged in a lattice configuration, they act as coupled dipoles and exchange energy. Figure 3b illustrates the generation of LSPR and SPPs at a single slot structure (top) and subsequent creation of SLR at the slot lattice (bottom) as a consequence of their interaction. Specifically, when a single slot antenna is optically excited, LSPR induces field localization at the slot, while SPPs launched from the slot propagate and decay within a limited range^29^. In contrast, when the slot antennas are periodically arranged and optically excited, the counter-propagating SPPs launched at different slots constructively interfere on the lattice plane, forming a 2D standing wave of SPPs^30^. When the anti-nodes of the resulting standing wave mode are periodically matched to the LSPR spots (slots), the coupling between SPPs and LSPR gives rise to a large-area field enhancement over the slot lattice, known as the SLR effect^31–33^. Figure 3c shows the simulated optical field distribution on the slot lattice, demonstrating the coherent non-local field enhancement by SLR, differentiated from the field localization in the single antennas (Fig. 2a, b).

In the presence of SLR, the polarizability of each antenna is significantly increased, resulting in an effective polarizability $\alpha$_eff_ = $1/(1/\alpha -S)$^34,35^. Here, $\alpha$ represents the polarizability of an individual antenna, and *S* denotes the dipole sum, which is determined by the cumulative contribution from surrounding antennas, including their respective distances, angles, and orientations, in addition to the incident optical field. As $\alpha$_eff_ increases, the extinction cross-section also increases, giving rise to enhanced light-matter interaction across a wide range^34,35^. To determine the optimal $\alpha$_eff_ inducing resonant coupling between the lattice and the MoSe_2_ ML, we calculate the field intensity (|*E*_*x*_|^2^) as functions of the period (*p*) and wavelength ($\lambda$) for slot and rod lattices, as shown in Fig. 3d, e (see SI Section 2 for length- and thickness-dependent field intensity and transmittance spectra for slot and rod lattices). In lattice structures, Rayleigh’s anomalies (RAs) also significantly influence SLR properties^36,37^. We indicate the plasmon resonance (black line) and RAs (white lines), ${\lambda }_{(n,{m})}=\frac{{n}_{d}}{\sqrt{{n}^{2}+{m}^{2}}}p$, where ${n}_{d}$ is the refractive index of a medium (${n}_{d}$ = 1) and (*n, m*) are integers defining the diffraction order, in the contour plots of Fig. 3d, e. Through strong coupling between plasmons and RAs, spectrally detuned *p*-dependent SLR energies are observed^38,39^ as shown in Fig. 3d. This resonance splitting behavior is not observed for the complementary rod lattice due to the weak interaction between the plasmons and RAs (Fig. 3e). We attribute the strong SLR feature in the slot lattice to the interference of SPPs on the Au surface, a factor absents in the rod lattice, indicating the advantage of this platform for enhancing PL signals of 2D vdWMs in a large area.

We then calculate dispersions of the slot lattice (*p* = 400 nm), exhibiting anti-crossing curves resulting from the interaction between two dispersive SPPs and a non-dispersive LSPR, as shown in Fig. 3f (see SI Section 3 for details of the model). This expected feature is well-measured in our angle-resolved reflectance spectroscopy experiment, as shown in Fig. 3g. Note that a ~1 nm thick polycarbonate film coated on the slot lattice to reduce the PL quenching of the MoSe_2_ ML causes a slight redshift of the lattice resonance (see SI Section 4 for more details). A thicker dielectric layer would more effectively prevent PL quenching. However, the primary focus of our work is to investigate the coupling properties between SLR and excitons, even though there is PL quenching, we employ a thin dielectric layer. This observed angle dependency is clear evidence of the induced SLR in our fabricated slot lattice (see SI section 5 for more results of control experiments). Note that a dark mode emerges at *k* = 0 with an energy of ~1.9 eV, probably originating from the non-radiative and coherent nature of bound states in the continuum (BIC) in the nanostructure^40^. We believe that exploring BIC-PL coupling would be an interesting direction for future study. Figure 3h shows the reflectance spectrum at *k*_*x*_*/k* = 0.38, extracted from Fig. 3g. The narrow linewidth of SLR (~65 meV), compared to the linewidth of LSPR (generally >200 meV), indicates the low-loss plasmonic system, in terms of damping, dephasing, and absorption^41–43^. Thus, the 2D vdWM-slot lattice hybrid can address the existing challenges associated with low-Q plasmonic systems while facilitating enhanced light-matter interactions in a large area.

### Large-area ultrathin 2D luminescent sheet enabled by surface lattice resonance

**Fig. 4:**
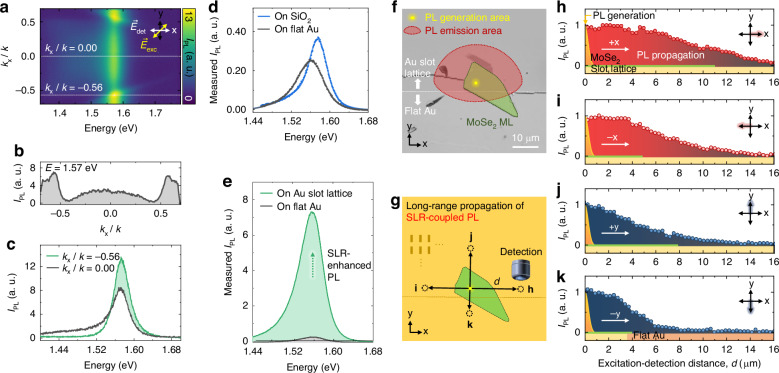
SLR-induced wide-range PL propagation. **a** Measured angle-resolved PL spectra of the slot lattice-coupled MoSe_2_ ML along *k*_*x*_ direction. **b** PL peak intensity at *E* = 1.57 eV as a function of *k*_*x*_*/k*, derived from (**a**). **c** PL spectra at different *k*_*x*_*/k*, derived from (**a**). **d** Measured PL spectra of the MoSe_2_ ML on the SiO_2_ (blue) and the Au (gray) substrates. **e** Measured PL spectra of the MoSe_2_ ML on the Au slot lattice (green) and at the Au surface (gray). **f** Optical microscopy image of the MoSe_2_ ML transferred onto the Au slot lattice, with indicated areas of PL generation (yellow) and emission (red). **g** Experimental scheme for the long-range PL propagation induced by SLR. **h**–**k** Measured PL peak intensity as a function of distance *d* along the $\pm$*x* and $\pm$*y* axes, from the excitation to the detection spot

Figure 4a shows the angle-resolved PL spectra of the MoSe_2_ ML on the Au slot lattice, measured along the *k*_*x*_ direction, exhibiting emission behaviors affected by SLR (see SI Section 6 for *k*_*y*_-resolved PL spectra). In this measurement, excitation polarization (*E*_exc_) is aligned along the diagonal axis, while detection polarization (*E*_det_) is along the *x* or *y* axis, as shown in Fig. 4a. At *k*_*x*_*/k* = $\pm$0.56, the SLR matches with the PL peak energy (1.57 eV), resulting in significant PL enhancement, as shown in Fig. 4a–c. On the other hand, in the angle-dependent PL measurement along the *k*_*y*_ direction, a slightly weaker PL enhancement is observed at *k*_*y*_*/k* = $\pm$0.35 (Fig. S6). This difference arises from the geometric anisotropy of the slot lattice, leading to different standing wave modes of SPPs in the *x* and *y* directions. Furthermore, LSPR of the slot lattice is more effectively induced along the *x*-axis, facilitating efficient LSPR-SPP coupling and resulting in substantial PL enhancement.

SLR-induced PL enhancement is affirmed through experimental comparison of PL spectra of MoSe_2_ ML on SiO_2_, at Au, and Au slot lattice. As shown in Fig. 4d, PL of MoSe_2_ ML on Au substrate typically undergoes intensity reduction, peak redshift, and linewidth broadening compared to MoSe_2_ on a SiO_2_ substrate due to the following reasons: enhanced non-radiative energy transfer, changes in the dielectric environment, charge transfer, and possible strain effects. Despite these collective factors modifying the optical properties, emitted PL coupled with SLR shows ~32-fold enhancement compared to the PL on the Au surface, as shown in Fig. 4e.

The standout characteristic of the 2D vdWM-slot lattice hybrid is the remarkable long-range propagation of the emitted PL across a substantial 2D area enabled by SLR. In our sample configuration, half of the MoSe_2_ ML resides on the Au slot lattice, while the other half rests on the flat Au surface, as shown in Fig. 4f (See SI Section 7 for optical microscopy and atomic force microscopy images of the sample). To quantify the propagation length of the emitted PL, we maintain a fixed local excitation spot (PL generation area in Fig. 4f) on the sample and measure the PL signal by scanning the detection lens as a function of distance (*d*) from the excitation spot along the $\pm$*x* and $\pm$*y* axes, as illustrated in Fig. 4g. The local PL generation area is estimated to be ~550 nm in diameter, using a high NA (0.8) objective lens. The spatial resolution of the detection setup is also ~550 nm, employing the same objective lens with confocal optics. Typically, in-plane excitons in 2D semiconductors exhibit short diffusion length with an emission direction perpendicular to the 2D surface^44–47^. On the other hand, the emitted PL coupled with SLR propagates across the 2D plane with distinct directionality, as also confirmed in the angle-resolved PL measurement (Fig. 4a). Therefore, we can significantly extend the PL emission area in our 2D vdWM-slot lattice hybrid.

Figure 4h–k shows the measured PL intensity as a function of distance *d* along the $\pm {x}$ and $\pm {y}$ axes from the PL generation area spot (See SI Section 8 for measured PL spectra as functions of distance *d*). The strong SLR effect in our slot lattice with the dominant resonance characteristics along the *x*-axis (Fig. 4h, i) facilitates longer PL propagation lengths compared to those along the *y*-axis (Fig. 4j, k). For comparison, we also performed the same propagation experiment with the MoSe_2_ ML on an Au substrate (SI Section 9). Notably, we suggest that a small piece of vdWM, approximately at the diffraction limit, may be used instead of a larger-size crystal to achieve this 2D luminescent sheet. This is supported by our findings, which demonstrate that the emitted PL coupled with SLR can propagate over long distances on the slot lattice, even in the absence of the MoSe_2_ ML (Fig. 4i).

In addition, PL propagation is observed on the flat Au surface without the slot lattice, extending up to several micrometers, as shown in Fig. 4k. Based on our experimental data, we estimate and indicate the PL emission area in the optical microscopy image (Fig. 4f). Assuming full coverage of the slot lattice, the emission area is ~800 μm^2^, calculated using (*d*_prop_)^2^, where *d*_prop_ represents the measured PL propagation distance along +*x* axis. This represents a new type of 2D luminescence device covering a large area, with local excitation at the diffraction-limit scale. Notably, achieving this 2D luminescent sheet does not require a large-sized 2D semiconductor. Instead, a small piece of vdWM crystal, approximately at the diffraction limit, suffices since this unique feature is facilitated by the long-range propagation of emitted PL on the slot lattice. This platform has the potential to be utilized in energy-efficient 2D illumination devices suitable for integration into portable electronics, such as smartphones and smartwatches. Multiple colors or white light illumination in these devices can be easily achieved using various pieces of vdWM crystals. In addition, by designing and fabricating diverse slot lattice structures, a range of optical devices, e.g., waveguides, splitters and couplers, multiplexers, and focusing devices^48–51^, can be fabricated, enabling optical integrated circuits and communications. Another intriguing aspect of our device is the ability to control the ballistic transport of electrically-neutral photons, facilitated by the directionally propagating characteristics induced by SLR. Consequently, this approach has the potential to address heating problems in electronic devices, particularly in this era of semiconductor miniaturization, by substituting electrons with non-electrically controllable photons. Furthermore, by utilizing the generated luminescent sheet, it becomes feasible to engineer expansive 2D fluorescence resonance energy transfer (FRET) devices tailored for swift chemical and biomedical sensing applications^52,53^.

## Discussion

In summary, we achieve a ~800 μm^2^ 2D luminescent sheet enabled by an Au slot lattice vdWM hybrid. By converting a rod antenna into an inverted slot antenna, we address the challenges of plasmonic antenna coupled vdWMs, specifically reduced ohmic loss and non-radiative decay enhancement. We then scale up to the slot lattice to induce the non-local photo-excitation of the coupled MoSe_2_ ML and long-range PL propagation. We validate the high in-plane momentum of the emitted PL coupled with SLR, and the extended PL emission area is measured as ~800 μm^2^, even beyond the MoSe_2_ ML flake. This work provides a significant step towards the next-generation optoelectronic devices, not only limited to ultra-bright wide displays, but potentially extended to quantum optical transportation applications with long coherence time and distance by coupling with optical communication devices.

## Materials and methods

### Fabrication of the Au slot and rod antenna lattices

The rod antenna was fabricated by using a high-resolution electron beam lithography (EBL) system on a SiO_2_ substrate. First, the 250 nm-thick MMA (Microchem, MMA (8.5) MAA EL-8) positive tone resist was spin-coated (5000 rpm, 60 s) on the SiO_2_ substrate and baked at 150 °C for 5 min on the hotplate. Then, the 60 nm-thick PMMA (Microchem, 950 PMMA A2) positive tone resist was spin-coated (2000 rpm, 60 s) on the MMA layer and baked at 180 °C for 5 min on the hotplate, leaving the MMA/PMMA bilayer structure. Using the EBL system (Elionix ELS-7800, acceleration voltage: 100 kV, beam current: 100 pA), the designed rod antenna array was exposed on the MMA/PMMA bilayer with respective different development rates to define a clear deposition area by a T-shaped profile after a subsequent development process. The MMA/PMMA bilayer was developed in MIBK:IPA = 1:3 solutions at 4 °C for 20 min. After being rinsed with IPA for 30 s and blown with air, the developed patterns were deposited with Cr (3 nm) and Au (30 nm) by an electron beam evaporation (KVT KVE-ENS4004), followed by the standard lift-off process. The slot antenna was fabricated by using a high-resolution focused ion beam (FIB) milling system on a 30 nm-thick Au layer, which is ready on the SiO_2_ substrate. First, 30 nm Au was deposited on the SiO_2_ substrate by the electron beam evaporation system (KVT KVE-ENS4004). Then, the FIB milling system (Helios G3 CX, acceleration voltage: 5 kV, beam current: 1 pA) carved slot antenna arrays on the Au layer by drawing lines with the length of the designed slot antenna.

### Fabrication of the MoSe_2_ ML-plasmonic structures hybrids

The hybrid of the MoSe_2_ ML and the Au slot antenna lattice is prepared with a polyethylene terephthalate (PET) stamp through the dry transfer technique. The thickness of the MoSe_2_ ML crystal is identified by the optical contrast of a microscope image, followed by the detailed spectroscopic characterization. The MoSe_2_ ML is exfoliated onto a silicon substrate with a 90 nm oxide layer. To fabricate the MoSe_2_ ML on the slot lattice, we employ the polyethylene terephthalate (PET) stamp to pick up the MoSe_2_ ML, ensuring accurate alignment using an optical microscope. The PET stamp with the MoSe_2_ ML is then stamped onto the prepared Au slot lattice. The polymer and samples are heated up to 70 °C for the pick-up and 130 °C for the stamp process, respectively. Finally, the PET is dissolved in dichloromethane for 12 h at room temperature.

### Angle-resolved reflectance and PL spectroscopy setup

For angle-resolved reflectance measurements, the sample was illuminated by white light from a halogen lamp (AvaLight-HAL-S-mini, Avantes) through an infinity-corrected 50$\times$ microscope objective with NA 0.65 (Plan Apo NIR HR, Mitutoyo). Reflected spectra were collected through the same objective in the back-reflection geometry. The back focal plane of the objective was projected onto the entrance slit of a spectrometer coupled to a liquid nitrogen cooled CCD camera (Princeton Instruments SP2500+PyLoN), which allowed simultaneous registration of reflected spectra for all angles within the collected NA. A spatial filter based on a rectangular double-slit mask in a 4f optical scheme was used to select light reflected from the Au lattice array and block unwanted background. Polarization was selected via thin-film polarizers placed in the excitation and detection channels. For PL measurements, the sample was illuminated by 632.8 nm HeNe laser, and angle-resolved spectra were collected in the same setup with an additional long pass filter (FELH0650, Thorlabs) placed in front of the spectrometer.

### PL propagation length measurement setup

The excitation laser (594 nm) was focused onto the sample using a bottom illumination scheme with a 100$\times$ objective lens (NA 0.8). The excitation polarization was set diagonally relative to the slot antenna’s short (*x*) and long (*y*) axes. PL responses were collected at the opposite side of the sample by a 100$\times$ objective lens (NA 0.8). The objective lens for the collection was scanned in the *x* and *y* directions by piezoelectric inertia actuators (Thorlabs, PIA25) while maintaining the focal length from the sample surface. The PL signals were then directed through a confocal setup and coupled to a multimode fiber to transmit signals to a spectrometer (f = 328 mm, Kymera 328i, Andor). An edge filter (600 nm cut off) was used in the detection line to remove the fundamental laser. The signals were finally imaged on a thermoelectrically cooled charge-coupled device (CCD, iDus 420, Andor) to obtain PL spectra.

## Supplementary information

Supporting information final

## Data Availability

The data that support the findings of this study are available from the corresponding author upon reasonable request.
