# Supplementary material for: High momentum two-dimensional propagation of emitted photoluminescence coupled with surface lattice resonance
Source: Light Sci Appl. 2025 Jun 20;14:218. doi: 10.1038/s41377-025-01873-3 (PMC12181263; doi:10.1038/s41377-025-01873-3)
Supplement: Supplementary file 1 — Supporting information final [file 41377_2025_1873_MOESM1_ESM.pdf]

Supplementary Information

High momentum two-dimensional propagation of emitted photoluminescence coupled with surface lattice resonance

Yeonjeong Koo[1†], Dong Kyo Oh[2†], Jungho Mun[2, 3], Artem N. Abramov[4], Mikhail Tyugaev[4], Yong Bin Kim[1], Inki Kim[5, 6], Tae Ho Kim[7], Sera Yang[7], Yeseul Kim[2], Jonghwan Kim[1, 7,8], Vasily Kravtsov*[4], Junsuk Rho*[2,9,10,11,12,13], and Kyoung-Duck Park*[1,9,14]

[1]Department of Physics, Pohang University of Science and Technology (POSTECH), 37673, Republic of Korea

[2]Department of Mechanical Engineering, Pohang University of Science and Technology (POSTECH), 37673, Republic of Korea

[3]Department of Electrical and Computer Engineering, Purdue University, IN 47907, USA

[4]School of Physics and Engineering, ITMO University, 197101, Russia

[5]Department of Biophysics, Institute of Quantum Biophysics, Sungkyunkwan University, 16419, Republic of Korea

[6]Department of Intelligent Precision Healthcare Convergence, Sungkyunkwan University, 16419, Republic of Korea

[7]Department of Material Science and Engineering, Pohang University of Science and Technology (POSTECH), 37673, Republic of Korea

[8]Center for Van der Waals Quantum Solids, Institute for Basic Science (IBS), 37673, Republic of Korea

[9]Institute for Convergence Research and Education in Advanced Technology, Yonsei University, 03722, Republic of Korea

[10]Department of Chemical Engineering, Pohang University of Science and Technology (POSTECH), 37673, Republic of Korea

[11]Department of Electrical Engineering, Pohang University of Science and Technology (POSTECH), 37673, Republic of Korea

[12]POSCO-POSTECH-RIST Convergence Research Center for Flat Optics and Metaphotonics, 37673, Republic of Korea

[13]National Institute of Nanomaterials Technology (NINT), 37673, Republic of Korea

[14]Department of Semiconductor Engineering, Pohang University of Science and Technology (POSTECH), 37673, Republic of Korea

*Email: vasily.kravtsov@metalab.ifmo.ru, jsrho@postech.ac.kr, parklab@postech.ac.kr

†These authors equally contributed to this work

1. Non-radiative decay rate maps.

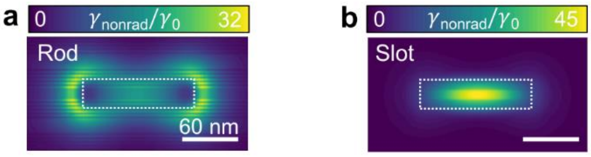

Fig. S1. a, b. Simulated distribution of nonradiative decay rate enhancement (γ_{nonrad}/γ_0) for the in-plane emitters distributed at the rod (a) and slot (b) antenna.

2. Parametric study of optical properties for rod and slot lattices.

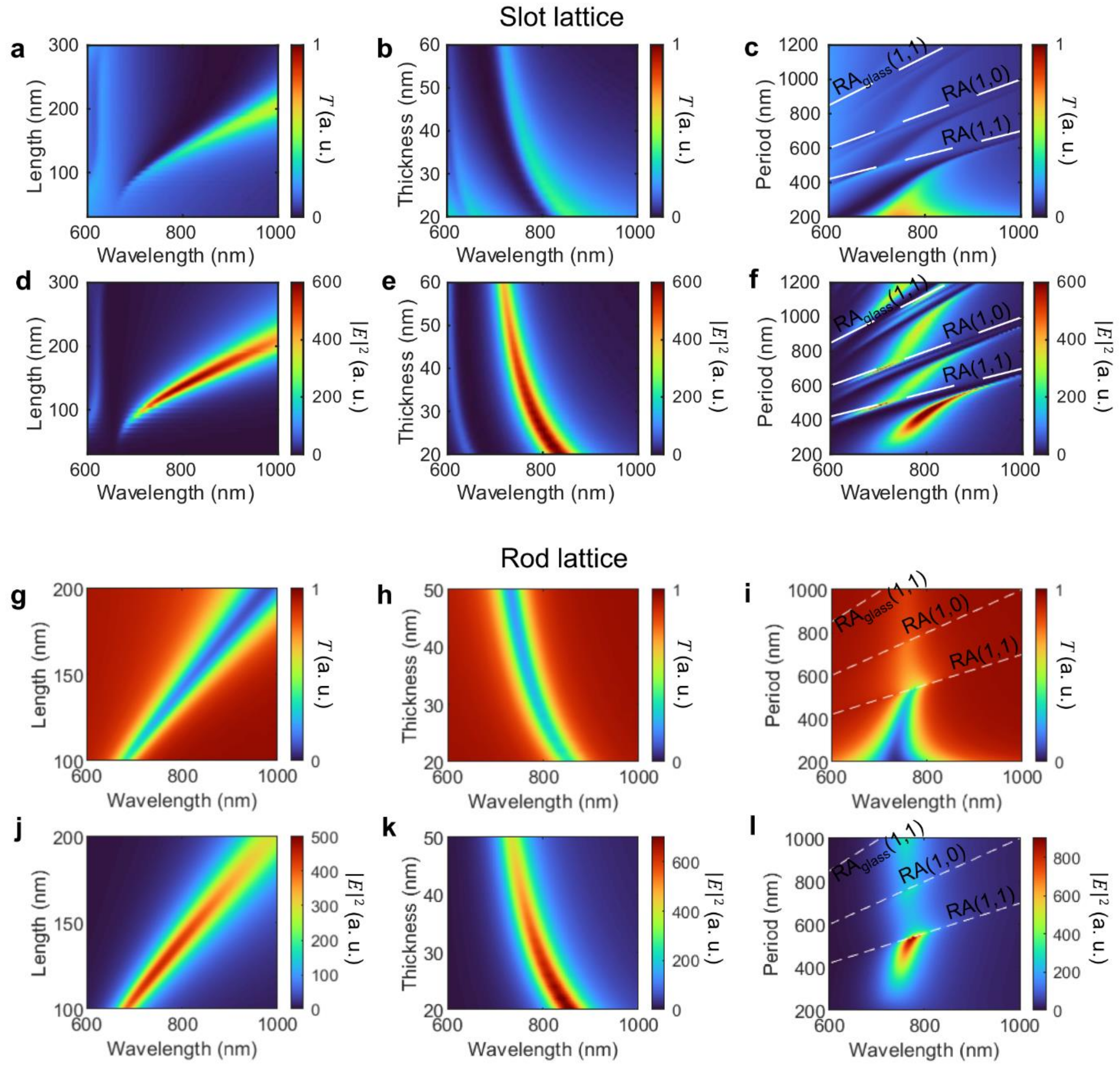

Fig. S2. Calculated transmittance (a-c, g-i) and field enhancement (d-f, j-l) spectra with changing length (a, d, g, j), thickness (b, e, h, k), and period (c, f, i, l) of the slot and the rod antenna lattice.

We conducted parametric studies by varying the length, the thickness, and period of the rod and the slot lattice. In general, the rod and slot lattice show consistent trends in optical responses as parameters changed, agreeing with Babinet's principle with subtle deviations. However, distinct behavior emerges in the rod and slot lattice concerning Rayleigh anomaly coupling in period-dependent transmittance (Fig. 2S c, i) and field enhancement (Fig. 2S f, l) contour plots.

Notably, the slot lattice exhibits a more pronounced dependency on the period for both transmittance and field enhancement, indicating stronger interaction between neighboring slots compared to the interaction between rods.

3. LSPR-counter propagating SPP coupling model.

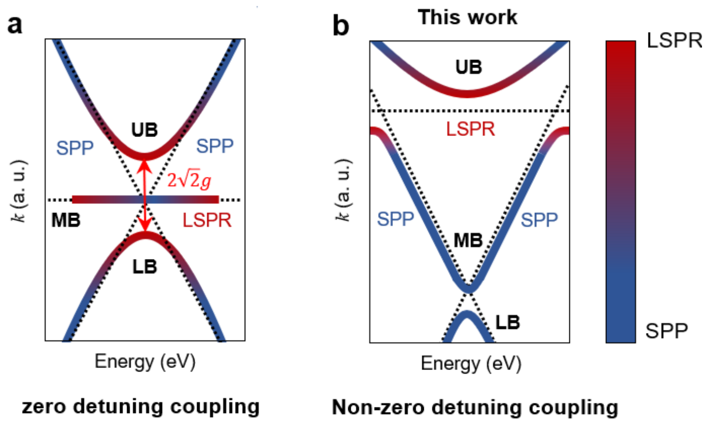

Fig. S3. Schematic diagram of the coupling dispersion for zero detuning (a) and non-zero detuning (b) between LSPR and SPP.

The coupling of LSPR and two counter-propagating SPPs of the slot lattice system can be expressed as a 3×3 matrix Hamiltonian.

$$H = \begin{pmatrix} E_{\mathrm{SPP1}} - \frac{i\gamma_{\mathrm{SPP1}}}{2} & g & g_{\mathrm{SPP-SPP}} \\ g & E_{\mathrm{LSPR}} - \frac{i\gamma_{\mathrm{LSPR}}}{2} & g \\ g_{\mathrm{SPP-SPP}} & g & E_{\mathrm{SPP2}} - \frac{i\gamma_{\mathrm{SPP2}}}{2} \end{pmatrix}$$

, where E_{SPP}, E_{LSPR} represent the energies of SPP and LSPR, respectively, SPP1 and SPP2 represent two counter-propagating SPPs, γ_{SPP} and γ_{LSPR} denote the decay rates, i.e., the spectral linewidths of the SPP or LSPR, respectively, g is the coupling strength between SPP and LSPR, and $g_{\mathrm{SPP-SPP}}$ is the coupling strength between two SPPs. Here, we consider the nearly linear dispersion of the propagating SPP, i.e., $E_{\mathrm{SPP}}(k)=\hbar vk$ (v is the velocity of SPP propagation) and the dispersionless LSPR. The SPP-SPP coupling strength $g_{\mathrm{SPP-SPP}}$ in the Hamiltonian is very weak compared to g, so it can be approximated to zero. Thus, all significant interactions between light and matter are determined by the coupling g between LSPR and SPP. If the two SPPs intersect at the same plasmon momentum (E_{SPP1} = E_{SPP2}), the eigenvalues are expressed as follows with the ignored damping

$$E_{U,L} = \frac{E_{SPP}+E_{LSPR}}{2} \pm \frac{1}{2}\sqrt{(E_{SPP} - E_{LSPR})^2 + 8g^2},$$

$$E_M = E_{SPP}$$

Here, E_{U}, E_{L}, and E_{M} represent the upper branch (UB), lower branch (LB), and middle branch (MB), of the resulted detuning $E(k)$ curves respectively. In the case of zero detuning coupling ($E_{\mathrm{SPP}} \cong E_{\mathrm{LSPR}}$), the E_{U} and E_{L} are split by $2\sqrt{2}g$, with an emerging MB between them (Fig. S3a). However, in this work, we are dealing with a non-zero detuning coupling system ($E_{\mathrm{SPP}} \neq E_{\mathrm{LSPR}}$), representing different dispersions from zero detuning. As a result, even with close-to-zero SPP-SPP coupling, mutual coupling with the LSPR leads to the effective coupling between the SPP branches, forming a parabolic SPP band structure (Fig. S3b). Our experimental results confirmed alignment with the non-zero detuning coupling model (Fig. 3g). Unfortunately, due to being outside the measurement range, we couldn't properly calculate g for strong coupling confirmation, so the coupling strength will not be mentioned quantitatively.

4. Angle-resolved reflectance spectra for the surrounding refractive index optimization.

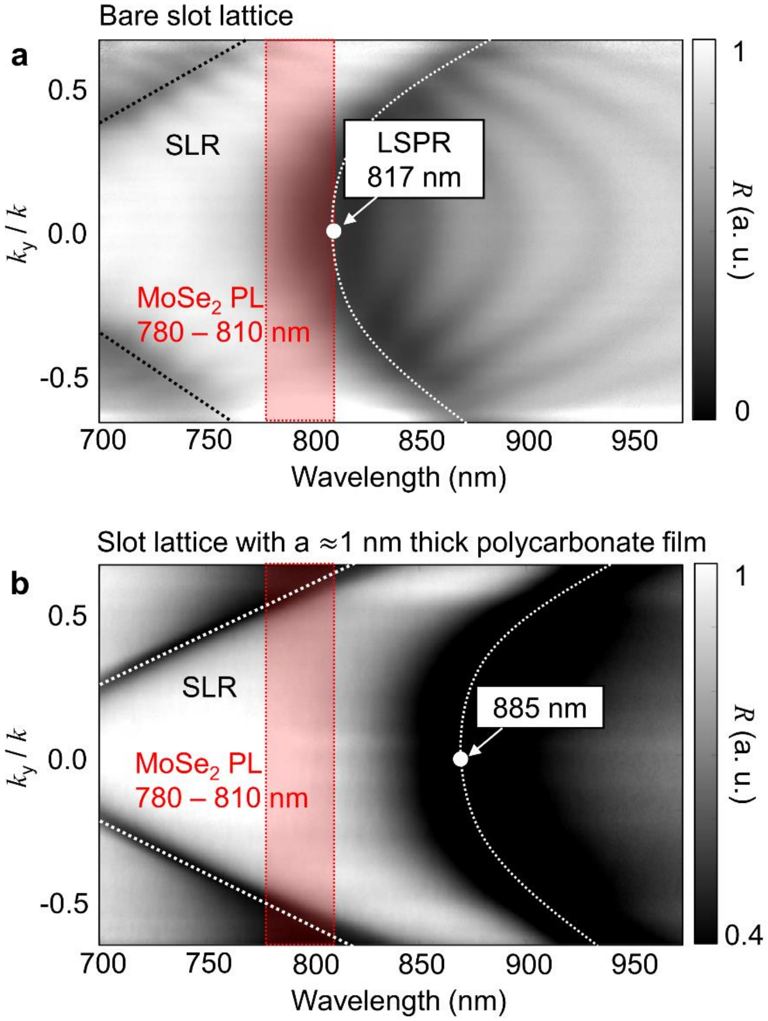

Fig. S4. Resonance shift of the angle-resolved reflectance spectra of a slot lattice before (a) and after (b) coating the polycarbonate film on the slot lattice. LSPR dip wavelengths at $k_y = 0$ are marked to check the resonance shift.

The optical response of a plasmonic structure to changes in refractive index (RIU) of the surrounding medium is quantified by its refractive index sensitivity (nm/RIU). In our study, we coat a polycarbonate film with a thickness of ≈1 nm onto a slot lattice with a refractive index of 1.59, resulting in a 68 nm redshift in the LSPR, in present of the SLR coupling. The refractive index sensitivity of our sample is calculated as 118.6 nm/RIU, a value consistent with the typical range for LSPR and SLR, falling between 100~450 nm/RIU.

5. Angle-resolved reflectance and PL spectra of slot lattice with different detection configuration.

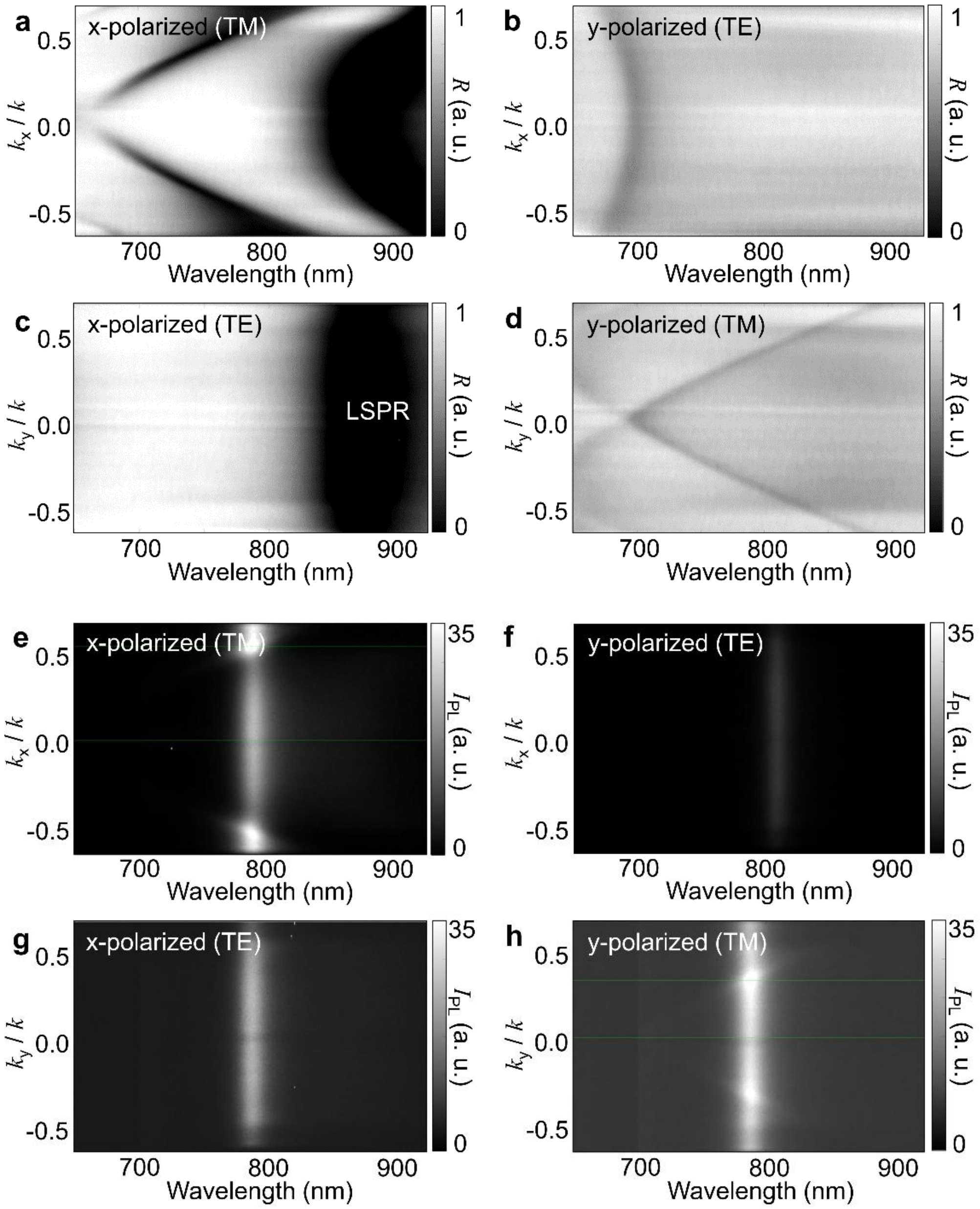

Fig. S5. Angle-resolved reflectance (a-d) and PL (e-h) spectra with a slot lattice coupled $MoSe_2$ ML with different detection configuration. **a, e.** k_x-resolved, *x*-polarized, **b, f.** k_x-resolved, *y*-polarized, **c, g.** k_y-resolved, *x*-polarized, and **d, h.** k_y-resolved, *y*-polarized.

We use diagonal polarization for the excitation beam for the angle-resolved spectroscopy. Through varying detection polarization and resolving the *k*-direction, we explore distinct optical modes of the slot lattice, as evidenced by reflectance and PL spectra in four different configurations; k_x- and k_y-resolving and *x*- and *y*-polarized detection.

The gold surface between the slots significantly suppresses the transverse electric (TE) mode SLR. This suppression results from the strong absorption of diffracted light with TE polarization propagating along the metal surface. The intense absorption is attributed to the

excitation of currents in the conductive layer, which effectively suppresses the diffractive coupling of localized resonances by reducing the dipole sum *S*.

However, for the transverse magnetic (TM) mode, coupling occurs through diffracted beams with the electric field perpendicular to the metal surface. Consequently, the SLR feature is observable only with TM mode detection, as seen in Figs. S5a and d, while it is absent in Figs. S5b and c.

In Fig. S5c, the LSPR from slot lattice is presented, without SLR feature. In Fig. S5b, LSPR is not evident with the inactive polarization of the antenna. Note that LSPR without SLR not changes its peak position with the angle of detection in contrast to the shifting SLR.

The enhanced PL spectra obtained from four distinct detection schemes exhibit distinct features. The PL spectra, influenced by SLR coupling (Figs. S5e and h), display angle dependency, whereas the PL spectra without SLR coupling exhibit minimal change with varying detection angles. The latter spectra demonstrate a slight decrease in intensity at higher angles, aligning with the radiation intensity of the in-plane dipole. This decrease corresponds to the radiation intensity of the in-plane exciton in the monolayer $MoSe_2$.

6. k_y-resolved PL spectra of $MoSe_2$ ML on the Au slot lattice.

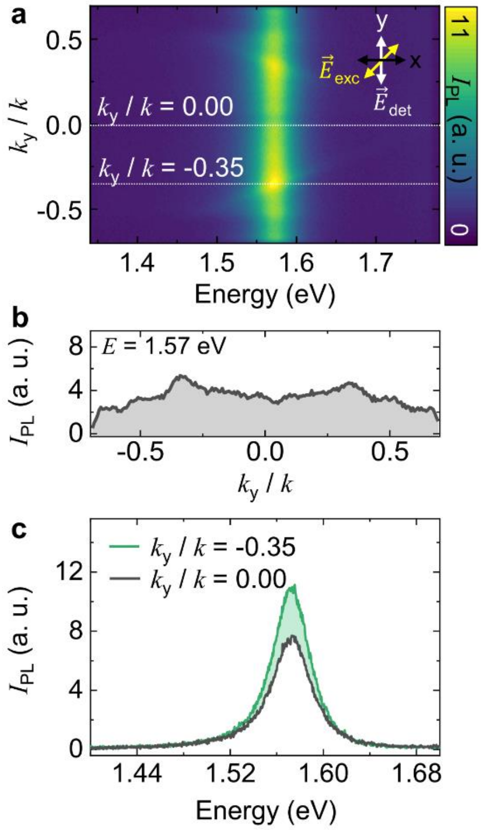

Fig. S6. a. Measured angle-resolved PL spectra of the slot lattice-coupled $MoSe_2$ ML along k_y direction. **b.** PL peak intensity at E = 1.57 eV as a function of k_y, derived from (**a**). **c.** PL spectra at different k_y, derived from (**a**).

The maximum PL intensity is acquired at $|k_y|$ = 0.35, at a smaller angle compared to the maximum PL intensity at $|k_x|$ = 0.56. This supports the results that emitted PL coupled with SLR propagates to a greater distance in the x-direction compared to the y-direction, as shown in Figs. 4h-k.

7. Optical microscopy and AFM topography images of the sample

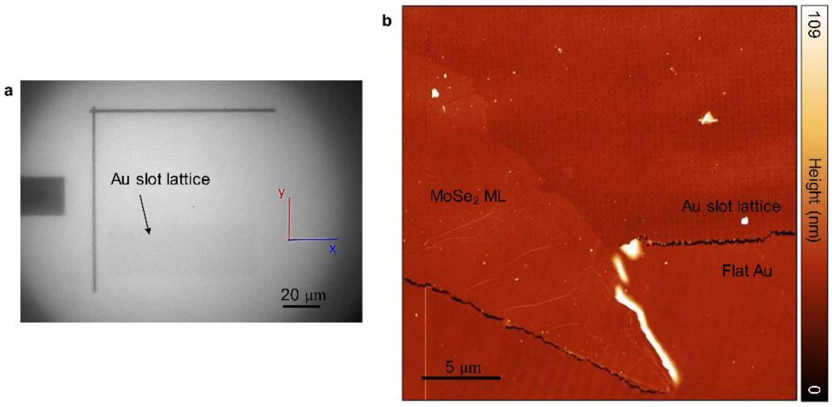

Fig. S7. An optical microscopy image (a, before the TMD monolayer transfer) and an AFM topography image (b, after the TMD monolayer transfer) of the sample.

8. Spatio-spectral map for the propagation of emitted PL coupled with SLR mode

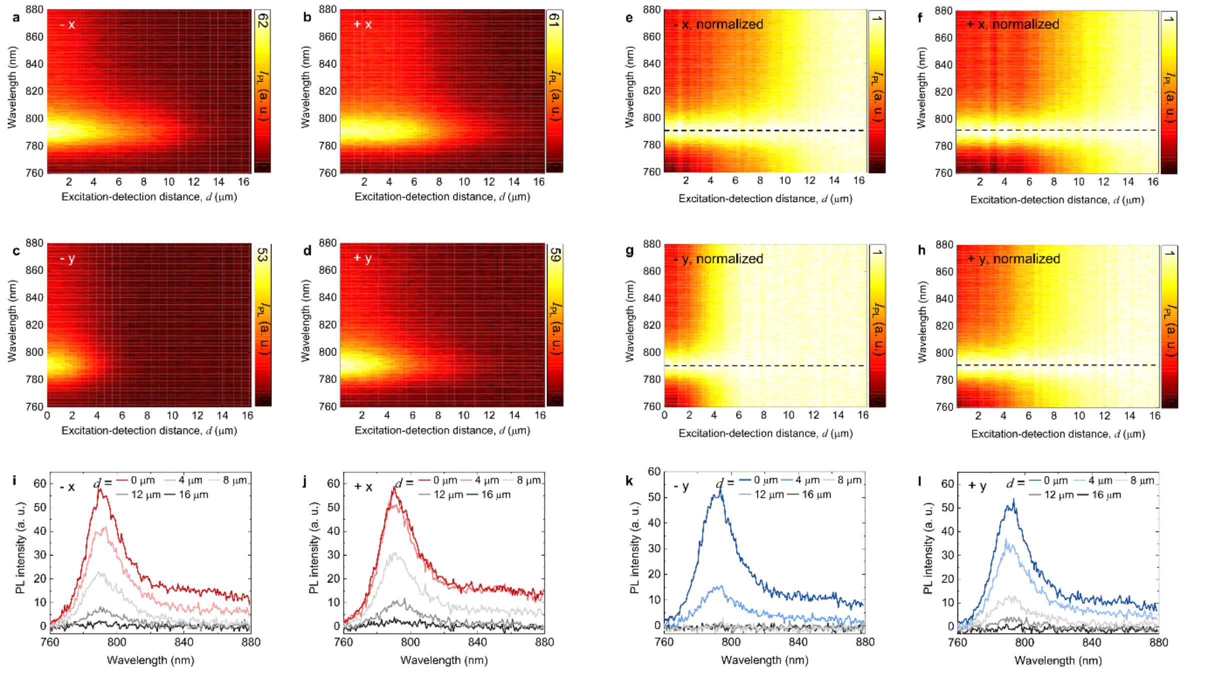

Fig. S8. a-d. Contour plot of measured PL spectra as functions of distance d along the $\pm x$ and $\pm y$ axes. **e-h.** Normalized PL spectra in a-d, respectively. **i-l.** PL spectra at the selected d = 0, 4, 8, 12, and 16 μm from a-d, respectively.

Fig. S8a-d present spatio-spectral maps for the propagation of emitted PL coupled with SLR mode as functions of propagation distance, d along the $\pm x$ and $\pm y$ axes, with corresponding normalized spectra (Fig. S8e-h). This confirms that the peak energy and linewidth of PL remain unchanged with d. Fig. S8i-l show the selected PL spectra extracted from Fig. S8a–d.

9. PL propagation on the flat Au substrate

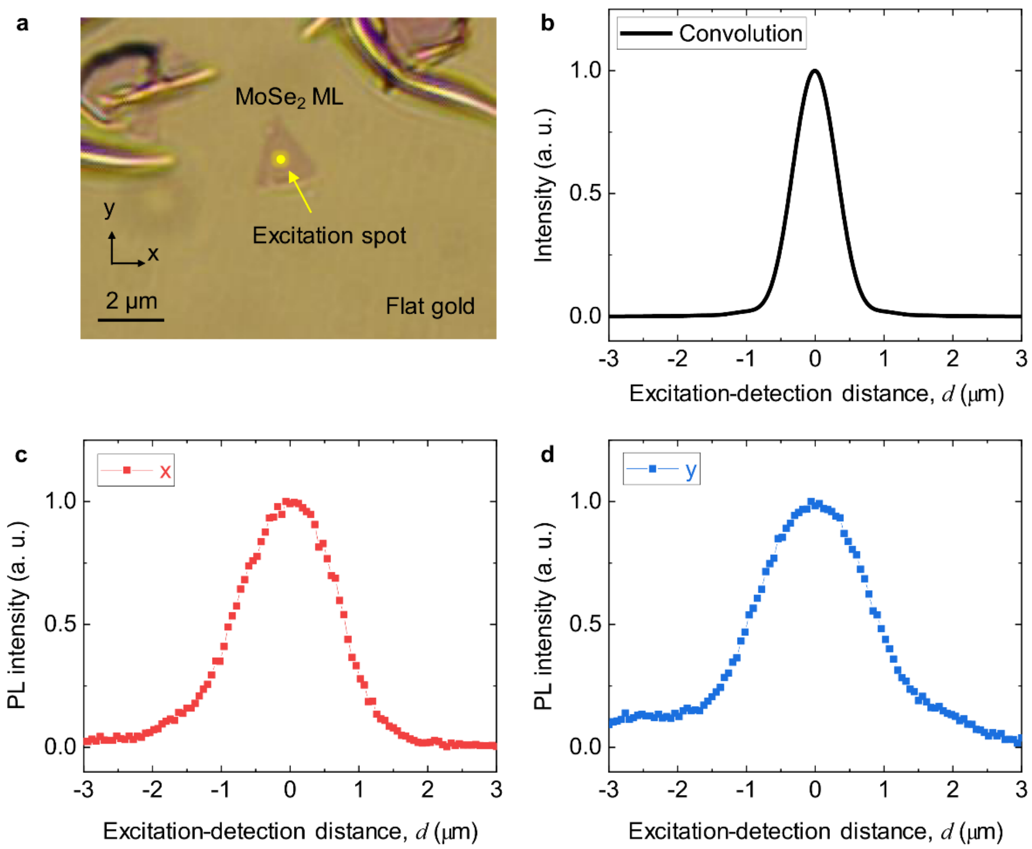

Fig. S9. a. An optical microscope image of a $MoSe_2$ ML on a flat gold substrate. **b.** Calculated convolution of fixed excitation spot and a movable detection spot. **c-d.** Measured PL intensity of a $MoSe_2$ ML on a flat gold substrate as a function of excitation-detection distance, d along the x (c) and y (d) axes.

We performed PL propagation measurements of a $MoSe_2$ ML transferred on a flat Au substrate (Fig. S9a). Without any SLR effects, the PL intensity profile can be modeled as a convolution of two Airy disks, corresponding to a fixed excitation spot and a movable detection spot. Under the experimental conditions, with both NA set to 0.8 and wavelengths of 594 nm and 795 nm, respectively, the theoretical intensity profile is shown in Fig. S9b. Similarly, the measured PL intensity profiles exhibit isotropic feature in $\pm$x and $\pm$y directions (c and d, respectively), closely matching the theoretical prediction. The slightly wider width is attributed to imperfections in the focused beam caused by lenses, such as aberrations.
